# New technologies in oral reconstructive medicine: Consensus report of the AAP/OF/SEPA Workshop

**DOI:** 10.1002/jper.70182

**Published:** 2026-09-14

**Authors:** Mariano Sanz, Sašo Ivanovski, José Nart, Frank Schwarz, Hom‐Lay Wang, Tara L. Aghaloo, Bilal Al‐Nawas, Gustavo Avila‐Ortiz, Shayan Barootchi, Juan Blanco, Gonzalo Blasi, Nikolaos Donos, Balazs Feher, Irina F. Dragan, Maria L. Geisinger, Adrián Guerrero, Effie Ioannidou, Darnell Kaigler, Yvonne Hernandez‐Kapila, Purnima S. Kumar, Liran Levin, Antonio Liñares, George A. Mandelaris, Paula Matesanz‐Pérez, Alberto Monje, Eduardo Montero, Alberto Ortiz‐Vigón, Joan Otomo‐Corgel, Flavia Q. Pirih, Ausra Ramanauskaite, Isabella Rocchietta, Vanessa Ruiz‐Magaz, Hanae Saito, Nerea Sánchez, Javier Sanz Esporrin, Anton Sculean, Florian M. Thieringer, Daniel S. Thoma, Cristina Valles, Diego Velásquez, David T. Wu, William V. Giannobile

**Affiliations:** ^1^ Faculty of Dentistry, ETEP Research Group Complutense University of Madrid Madrid Spain; ^2^ School of Dentistry The University of Queensland Brisbane Australia; ^3^ Department of Periodontology Universitat Internacional De Catalunya Barcelona Spain; ^4^ Department of Oral Surgery, Implantology and Oral Medicine Goethe University Frankfurt Frankfurt Germany; ^5^ Department of Periodontics and Oral Medicine University of Michigan School Of Dentistry Ann Arbor Michigan USA; ^6^ Section of Oral and Maxillofacial Surgery University of California Los Angeles School of Dentistry Los Angeles California USA; ^7^ Department of Oral and Maxillofacial Surgery Universitäts Medizin Mainz Johannes Gutenberg University Mainz Mainz Germany; ^8^ Department of Oral Medicine, Infection, and Immunity Harvard School of Dental Medicine Boston Massachusetts USA; ^9^ Department of Surgery and Medical‐Surgical Specialties Universidade de Santiago de Compostela Santiago de Compostela Spain; ^10^ Institute of Dentistry Queen Mary University of London London United Kingdom; ^11^ Department of Oral and Maxillofacial Surgery Harvard School of Dental Medicine Boston Massachusetts USA; ^12^ Department of Periodontology Tufts University School of Dental Medicine Boston Massachusetts USA; ^13^ Private Practice The Perio Studio Boston Massachusetts USA; ^14^ Department of Periodontology University of Alabama at Birmingham Birmingham Alabama USA; ^15^ Private Practice Clínica Guerrero Marbella Spain; ^16^ Division of Periodontology University of California San Francisco School of Dentistry San Francisco California USA; ^17^ Section of Periodontics, Division of Regenerative and Reconstructive Sciences University of California Los Angeles School of Dentistry Los Angeles California USA; ^18^ Bitonte College of Dentistry Northeast Ohio Medical University Rootstown Ohio USA; ^19^ Private Practice, Periodontics and Dental Implant Surgery Glenview and Oakbrook Terrace Illinois USA; ^20^ Arrow Development ARC Research Bilbao Spain; ^21^ Greater VA Healthcare System Dental Service Los Angeles California USA; ^22^ Department of Periodontology University College London Eastman Dental Institute London United Kingdom; ^23^ Division of Periodontics University of Maryland School of Dentistry Baltimore Maryland USA; ^24^ Department of Periodontology University of Bern Bern Switzerland; ^25^ Department of Oral and Maxillofacial Surgery University of Basel Basel Switzerland; ^26^ Clinic of Reconstructive Dentistry, Center of Dental Medicine University of Zurich Zurich Switzerland; ^27^ Department of Periodontology, Research Institute for Periodontal Regeneration Yonsei University College of Dentistry Seoul Republic of Korea

**Keywords:** biologics, cell therapy, dental implants, periodontology, regeneration, regenerative medicine, scaffolds

## Abstract

**Background:**

The objectives of this Workshop were to evaluate the efficacy of scaffolding technologies for horizontal and vertical ridge augmentation; to evaluate the efficacy of substitute scaffolds for soft tissue augmentation in the context of implant therapy; to evaluate the efficacy of cells and/or biologics combined with scaffolds for alveolar bone regeneration; to evaluate the efficacy of cells and/or biologics combined with scaffolds for soft tissue regeneration in the context of implant therapy; and to evaluate emerging technologies and personalized strategies for improving hard and/or soft tissue defects around natural teeth and dental implants.

**Methods:**

The Workshop discussions were informed by five specifically commissioned systematic reviews from the following three Working Groups: Working Group 1 – Use of scaffolds for bone and soft tissue regeneration; Working Group 2 – Use of cells and/or biologics combined with scaffolds for hard and soft tissue regeneration; and Working Group 3 – Current, emerging, and personalized approaches for periodontal and peri‐implant tissue regeneration. The Workshop participants were informed by the compiled evidence from the five systematic reviews within each Working Group to address pertinent issues to inform clinical practice, research, and the public community.

**Results:**

The consensus among the Workshop group participants was that cellular therapies and biologics, alone and in combination with scaffolding technologies, offer strong potential in regenerating tissues around teeth and dental implants. There was variability in treatment outcomes depending on the overall evidence and the efficacy of these technologies, as well as on the specific defects and patient‐based constraints. The consensus report detailed the results and implications of the findings, demonstrating the value of these technologies in improving clinical and patient‐reported outcomes.

**Conclusions:**

Current regenerative approaches lead to long‐term stable outcomes at natural teeth as well as dental implant sites; novel technologies can benefit patients by enhancing longer‐term stability of teeth and implants to promote the health of the oral cavity and dentition; and artificial intelligence–enabled modeling can contribute to the identification of relevant features at the individual (as opposed to population) level, empowering patients to be active participants in their journeys and improving science‐to‐public communication.

## Plain language summary

This publication reports the consensus from a Workshop that evaluated the efficacy of current and emerging technologies for bone regeneration and soft tissue augmentation in the context of dental implant therapy. Based on the three elements of tissue bioengineering (scaffolds, cells, and molecular mediators), purposefully dedicated systematic reviews evaluated evidence on the use of scaffolds alone or in combination with cells and biologics to reconstruct hard and soft tissues in the oral cavity. Based on this evidence, the experts gathered at the Workshop elaborated a consensus report describing the state of the art and emerging evidence for the use of these technologies in the context of both hard and soft tissue regeneration. The main conclusion of this report is that current regenerative approaches lead to long‐term, stable hard‐ and soft‐tissue outcomes, both when aimed at tissue reconstruction around natural teeth and at dental implant sites. Emerging technologies will benefit patients by enhancing these outcomes, thereby promoting oral health. Artificial intelligence–enabled modeling will help personalize these therapies, empower patients to be more actively engaged, and improve science‐to‐public communication.

## 1 INTRODUCTION

Oral and maxillofacial bone and soft tissue defects or deformities present a significant challenge for patients, often resulting from chronic inflammatory conditions such as periodontitis or peri‐implantitis, as well as following traumatic injury, tumor resection, or severe alveolar atrophy. Bone and soft tissue loss can significantly impair oral function, aesthetics, phonetics, and quality of life (QoL), as well as hinder oral rehabilitation with dental implants.

Specifically, oral and maxillofacial bone defects are primarily treated with surgical procedures that involve grafting materials, such as autologous, xenogeneic, or allogenic bone grafts, combined with barrier membranes, following the principles of guided tissue regeneration. However, using autologous grafts requires a second surgical site, which increases morbidity and complications. While allogenic and xenogeneic grafts are more readily available, they primarily serve as passive fillers and do not actively modulate inflammation, recruit progenitor cells, or promote vascularization—all of which are essential for stable and functional bone regeneration.

While current strategies for bone regeneration are effective for minor bony defects, they are limited when applied to more complex sites. In these situations, current regenerative methods remain invasive and often fail to reliably restore function and enable dental implant placement, leaving clinicians with few options and patients without effective, well‐accepted solutions. This challenge is especially critical for elderly or systemically compromised patients, who have reduced healing capacity and fewer treatment choices.

Soft‐tissue deformities affecting teeth and dental implants, whether occurring alone or in association with bone defects, are primarily attributable to trauma, aging, and/or chronic oral inflammatory conditions, especially in patients with limited soft‐tissue phenotypes, or to therapies such as orthodontics, which increase root prominence and reduce soft‐tissue thickness. These deformities have traditionally been managed with autografts, which require a second surgical site, usually the palate, increasing patient morbidity and complications. To address this issue, soft‐tissue graft substitutes have been developed, offering clear benefits such as unlimited availability and the absence of donor‐site morbidity. However, they have generally yielded inferior clinical outcomes.

To address these limitations, this consensus report, based on five systematic reviews, has aimed to evaluate the scientific evidence on the use of emerging technologies in bioengineered approaches, mainly involving innovative biomaterials as scaffolds, biologics, and cell therapies for adequate intraoral bone and soft tissue regeneration of defects around teeth and dental implants.

The specific objectives of this Workshop were:

- To evaluate the efficacy of scaffolding technologies for horizontal and vertical ridge augmentation.
- To evaluate the efficacy of substitute scaffolds for soft tissue augmentation (STA) in the context of implant therapy.
- To evaluate the efficacy of cells and/or biologics combined with scaffolds for alveolar bone regeneration.
- To evaluate the efficacy of cells and/or biologics combined with scaffolds for soft tissue regeneration in the context of implant therapy.
- To evaluate emerging technologies and patient‐specific strategies for improving hard and/or soft tissue defects around natural teeth and dental implants

## 2 MATERIALS AND METHODS

The Workshop on Novel Technologies in Oral Reconstructive Medicine was jointly organized by the American Academy of Periodontology (AAP), Osteology Foundation (OF), and the Sepa Foundation (SEPA). The in‐person meeting took place at the Parador de Alcalá de Henares (Spain), from September 28 to 30, 2025. A total of 42 experts examined the current evidence derived from five commissioned systematic reviews on the topic and debated their findings and implications.

The scope of the Workshop covered emerging strategies for oral tissue regeneration, including bone and soft tissue grafting (autologous, allografts, xenogeneic, synthetic), cell therapy and biologics combined with grafts, and personalized approaches and novel technologies to enhance regenerative outcomes. Five systematic reviews were commissioned to specifically address different groups of approaches: (i) scaffolds for bone regeneration; (ii) scaffolds for soft tissue regeneration; (iii) biologics and/or cells combined with scaffolds for bone regeneration; (iv) biologics and/or cells combined with scaffolds for soft tissue regeneration; and (v) digital and personalized workflows. Three Working Groups (WGs) were established to organize discussions and prepare the consensus report.

### 2.1 Workgroup 1 – Use of scaffolds for bone and soft tissue regeneration

Working Group 1, chaired by Frank Schwarz and José Nart, focused on the use of scaffolds for bone and soft tissue regeneration. The systematic reviews were led by Kumar, Donos, Avila‐Ortiz, and Jung.

### 2.2 Workgroup 2 – Use of cells and/or biologics combined with scaffolds for hard and soft tissue regeneration

Working Group 2, chaired by Mariano Sanz and Hom‐Lay Wang, focused on the use of biologics and/or cells combined with scaffolds for bone and soft tissue regeneration. The systematic reviews were led by Monje, Geisinger, Montero, and Mandelaris.

### 2.3 Workgroup 3 – Current, emerging, and personalized approaches for periodontal and peri‐implant tissue regeneration

Working Group 3, chaired by William Giannobile and Sašo Ivanovski, focused on emerging technologies and personalized strategies to improve the results of regenerative therapies. The systematic review was led by Kaigler and Barootchi.

## 3 CONSENSUS REPORT

### 3.1 Use of scaffolds for bone and soft tissue augmentation

In contemporary implant dentistry, bone augmentation and STA are often required to enable ideal implant placement, maximize esthetic and functional outcomes, and achieve long‐term stability and patient QoL. Traditionally, autogenous grafts have been considered the standard of care due to their biocompatibility and predictability, yet they are associated with several disadvantages, such as increased surgical complexity and postoperative morbidity.^1^, ^2^, ^3^ Engineered scaffolds and substitutes have been introduced to simplify procedures, reduce morbidity, and expand treatment options.

Two systematic reviews prepared for this consensus comprehensively addressed these approaches. Saleh et al.^4^ evaluated patient‐specific scaffolds for horizontal and vertical ridge augmentation, reporting substantial bone gains but also highlighting a considerable risk of complications, particularly soft tissue dehiscence and scaffold exposure. These outcomes point to a need for careful case selection and surgical expertise. In another systematic review, Avila‐Ortiz et al.^5^ comparatively assessed autogenous soft tissue grafts and matrices. Connective tissue grafts (CTGs) and free mucosal grafts remain as the reference standard for increasing mucosal thickness (MT) and keratinized mucosa, respectively, though substitute scaffolds such as collagen matrices and dermal allografts can provide comparable short‐term results in terms of MT gain, as well as reduced morbidity and lower postoperative discomfort. Together, these reviews underscore the importance of balancing efficacy with the potential patients’ risk of complications when selecting augmentation strategies.^6^

Based on this evidence and framework, Working Group 1 aimed to formulate key consensus questions defining and classifying materials and techniques for augmentation, identifying clinical indications for bone augmentation (horizontal,^6^ vertical, or combined ridge deficiencies to be treated simultaneously or in a stage approach manner; and contour corrections for esthetics and emergence profile^7^) and STA (peri‐implant keratinized tissue width augmentation,^8^ peri‐implant soft tissue volume augmentation,^8^, ^9^, ^10^ peri‐implant soft tissue dehiscences (PSTDs),^11^, ^12^ peri‐implant interproximal papilla reconstruction,^13^ discoloration/pigmentation of the peri‐implant mucosa^14^), establishing standardized outcome measures, evaluating efficacy, and addressing potential complications associated with STA and bone augmentation, while also highlighting priorities for future research and development.

#### 3.1.1 Efficacy of patient‐specific scaffolds for horizontal and vertical ridge augmentation

##### Definitions

For this consensus, the following definitions were adopted:

- *Engineered Scaffold*: A computer‐aided design and computer‐aided manufacturing (CAD/CAM) ‐customized, patient‐specific device designed to fit a defined ridge defect and maintain space for bone regeneration. It is fabricated as a block or framework, used alone or with bone replacement grafts, with or without cell therapy.
- *CAD/CAM Titanium Mesh*: A customized perforated titanium barrier produced by milling or selective laser melting/Direct Metal Laser Sintering (DMLS). It provides rigid space maintenance and can be fixated. It is often combined with particulate grafts and sometimes covered by a resorbable membrane.^15^
- *PEEK Shell*: A customized polyether‐ether‐ketone (PEEK) barrier or “shell” that creates and protects the regenerative space. It offers high fatigue resistance and favorable handling and is typically combined with particulate grafts.^16^
- *Customized Allogenic Bone Block*: A CAD/CAM‐milled allogenic bone block tailored to the defect to reduce intraoperative shaping and donor‐site morbidity; usually combined with fixation and particulate augmentation as needed.^17^
- *3D‐Printed HA/Other Printed Bone Blocks*: Additively manufactured, osteoconductive blocks (e.g., hydroxyapatite) shaped to the defect; used with or without adjunctive grafts or membranes.^18^
- *Barrier (Absorbable Membrane)*: An absorbable membrane optionally placed over the scaffold to support soft‐tissue management, graft containment, and compartmentalization.^19^
- *Protected Bone Healing/Space Maintenance*: This is the biologic and mechanical principle that describes a stable space that permits vascular ingrowth, cell migration, and bone formation while resisting soft tissue collapse and graft displacement during the healing period.^20^
- *Early Complications*: complications occurring < 4 weeks after surgery.^21^
- *Late Complications*: complications occurring ≥4 weeks after surgery.
- *Intraoperative Shaping*: Situations where chairside adaptation of standard meshes/blocks is required, and where a customized device may reduce operative time and variability.^22^
- *Prosthetically Guided Regeneration (PGR)*: This technique is used within a digital workflow to align augmentation with the planned implant position and prosthetic envelope.^23^

The following outcomes for assessing the efficacy of patient‐specific scaffolds for alveolar ridge augmentation were selected: Clinical Outcome Assessments^24^, ^25^ including (1) Clinician‐reported outcomes (ClinROs): (a) Clinical,^26^ (b) Esthetics,^27^, ^28^ (c) Safety; (2) Patient‐reported outcomes and experiences^29^ (PROs/PREs): (a) Satisfaction, (b) Morbidity,^30^ (c) Esthetics, (d) Function/comfort^31^; (3) Biomarkers, EndpointS, and other Tools (BEST) resources^32^: (a) Histomorphometry, (b) Bone quality/density, (c) Implant stability, (d) Radiographic.

The efficacy of patient‐specific scaffolds for alveolar ridge augmentation was assessed in relation to the following outcomes: dimensional gains,^33^ influence of membranes and materials,^7^, ^19^ modifiers (smoking, clinician experience, defect type^7^, ^34^), and bone quality/density and histology.

The consensus report was based on the answer to the following questions:

1. For horizontal ridge augmentation performed simultaneously with implant placement, what horizontal bone gain is achieved when using engineered scaffolds, and how does this outcome compare to that of guided bone regeneration (GBR) without custom scaffolds?
  For this question, no eligible studies were identified that performed horizontal ridge augmentation using an engineered scaffold simultaneously with implant placement.
2. For horizontal ridge augmentation performed in a staged approach prior to implant placement, what horizontal bone gain is achieved with the use of engineered scaffolds as compared to that of GBR without custom scaffolds?
  Based on the systematic review by Saleh et al., no direct comparison was performed across the three included studies. Overall, engineered scaffolds (with or without a membrane) achieved a horizontal gain of 4.96 mm (95% confidence interval [CI]: 4.48–5.44) at 4–10 months.^4^
3. For vertical ridge augmentation performed in a staged manner before implant placement, what vertical bone gain is achieved with engineered scaffolds versus GBR without custom scaffolds?
  Based on meta‐analysis by Saleh et al.,^4^ which included five studies (145 cases), engineered scaffolds achieved an overall vertical gain of 5.64 mm (95% CI 5.26–6.02) at approximately 6–9 months. A single, non‐inferiority randomized controlled trial (RCT) demonstrated no significant differences between customized CAD/CAM Ti‐mesh scaffold versus Ti‐reinforced dense polytetrafluoroethylene (d‐PTFE) barrier.^35^
4. For vertical ridge augmentation performed simultaneously with implant placement, what vertical bone gain is achieved using engineered scaffold‐based techniques in comparison to GBR without custom scaffolds?
  For this question, no eligible studies were identified that performed vertical ridge augmentation using an engineered scaffold simultaneously with implant placement.
5. For alveolar ridge augmentation, what volumetric bone gain is achieved with engineered scaffolds versus GBR without custom scaffolds?
  For this question, no eligible studies were identified that directly compared engineered scaffolds with GBR without custom scaffolds. Based on the systematic review by Saleh et al.^4^, customized CAD/CAM titanium mesh was used in five studies (83 cases). It resulted in an overall gain of 1307 mm^3^ (95% CI: 1000‐1700), which increased to 1715 mm^3^ (95% CI: 1200–2200) when an absorbable membrane was added (three studies, 51 cases).
  Furthermore, the volumetric gain was lower in the maxillary sites when compared to the mandibular sites. In univariate and multivariate analyses of volumetric bone gain, a combination of autogenous particles and xenograft was associated with increased volumetric bone gain, with an additional 745.3 mm^3^ (95% CI: 403.2–1087.3; *p* < 0.0001) in the multivariate analysis.
6. What are the early complication rates associated with using engineered scaffolds for alveolar ridge augmentation, and how do these compare to the complication rates observed with GBR without custom scaffolds?
  The overall rate of early complications for the engineered scaffolds was 21.7% (95% CI, 16.4–27.0). When an absorbable membrane was added (51 cases), a lower complication rate of 21.4% (95% CI 10.1–32.6%) was observed compared with 35.2% (95% CI 24.1–46.2%) when a resorbable membrane was not added (72 cases).
7. What are the late complication rates associated with using engineered scaffolds for alveolar ridge augmentation, and how do these compare to the complication rates observed with GBR without custom scaffolds?
  The overall rate of late complications for the engineered scaffolds was 19.8% (95% CI, 12.2–27.4). When an absorbable membrane was added, a lower complication rate of 11.1% (95% CI −9.4 – 31.6%) was observed in one study^36^ versus 52.9% (95% CI 29.2 – 76.7%) when an absorbable membrane was not included in another study.^37^
8. What do current data suggest about the long‐term stability of bone gained through engineered scaffold‐based augmentation?
  For this question, no eligible studies (≥ 5‐year follow‐up) were identified.
9. What patient‐related or defect‐related factors have been shown to affect the success, amount of bone gain, or risk of complications in scaffold‐based ridge augmentation?
  Light smoking was associated with less bone gain as compared to non‐smoking: horizontal −2.40 mm (95% CI −4.23 to −1.26; *p* = 0.031) and vertical −1.12 mm (95% CI −2.73 to −0.21; *p* = 0.001). Combined defects versus horizontal defects were associated with greater vertical gain (+3.11 mm) and higher volumetric gain (+720.13 mm^3^). Maxillary sites showed lower volumetric gain than mandibular sites (−26.02 mm and−21.57 mm^3^, respectively).
10. How does the level of surgical experience influence the outcomes following the use of engineered scaffolds for ridge augmentation?
  A meta‐regression analysis (10 studies) presented by Saleh et al.^4^ indicated 2.42 mm more horizontal bone gain when experienced surgeons performed the surgery compared to graduate students (95% CIs: 0.91‐5.48, p = 0.021).
11. What is known about PROs (such as intra‐ and post‐operative discomfort or satisfaction with treatment) following the use of engineered scaffolds for ridge augmentation?
  According to the meta‐analysis by Saleh et al., two studies reported PROs, each showing low–to–moderate pain on visual analog scale/numeric rating scale (VAS/NRS) during surgery and throughout follow‐up, regardless of the augmentation technique used.

#### 3.1.2 Efficacy of substitute scaffolds for soft tissue augmentation in implant therapy

##### Definitions

- *Autogenous Soft Tissue Graft*: Soft tissue of autogenous origin employed to treat mucogingival deformities via augmentation, repair, and/or regeneration. These soft tissues are transferred from one position to another within the same individual. There are two main categories of autogenous soft tissue grafts of intraoral origin: free gingival grafts and connective tissue grafts.
- *Free Gingival Graft (FGG)*: Also known as free mucosal graft, it is a portion of the oral mucosa that contains a keratinized epithelium layer and a portion of the underlying lamina propria.
- *Connective Tissue Graft (CTG)*: Also known as subepithelial connective tissue graft, it is a portion of the oral mucosa constituted by the lamina propria, submucosa, or a combination of both.
- *Soft Tissue Graft Substitute*: Any biomaterial of exogenous origin (i.e., allograft, xenograft, or alloplastic material) employed to treat a mucogingival deformity via augmentation, repair, and/or regeneration.
- *Allograft*: Also known as homograft, homologous, allogenous, or allogenic grafts, these are soft tissue graft alternatives obtained from a human donor.
- *Xenograft*: Also known as heterografts, xenogenous, or xenogeneic grafts, these are graft substitutes of non‐human origin.
- *Alloplastic Materials*: Also known as alloplasts, these are graft substitutes of synthetic origin.

The efficacy of autogenous grafts versus soft‐tissue substitute scaffolds in augmenting peri‐implant soft tissues was assessed as: (1) ClinROs. (a) Clinical: linear changes in peri‐implant keratinized mucosa width (KMW), MT, supracrestal tissue height (STH), marginal mucosa position, changes in peri‐implant mucosa volume, implant survival rate, and incidence of peri‐implant mucositis and peri‐implantitis. (b) Radiographic: marginal bone level changes. (c) Esthetics: Assessment using a standardized method (e.g., Pink Esthetic Score [PES]). (d) Safety: postoperative complications and adverse events. (2) PROs/PREs. (a) Overall satisfaction or perceived benefit (questionnaires, VAS, or NRS). (b) Esthetic perception assessed using questionnaires, VAS, or NRS. (c) Morbidity: postoperative pain or discomfort (VAS or NRS), and consumption of analgesics or painkillers.

The consensus report was based on the answer to the following questions:

1. What is the clinical performance of autogenous soft tissue grafts compared with substitute materials in terms of peri‐implant MT augmentation?
  At sites with insufficient MT, STA may be considered. Based on the results of a network meta‐analysis,^5^ which included data extracted from primary studies with a 6‐ to 12‐month follow‐up, a bilaminar approach involving the use of a subepithelial CTG, or an acellular dermal matrix represents the preferred treatment modalities. A qualitative assessment of the selected evidence showed that patients who received STA were equally or slightly more satisfied with their overall experience than patients randomized to the control groups.
2. What is the clinical performance of autogenous soft tissue grafts compared with substitute materials in terms of peri‐implant KMW augmentation? What surgical approach typically results in the greatest gain?
  In sites displaying an inadequate amount of KMW, STA may be considered. Based on the results of a network meta‐analysis,^5^ which included data extracted from primary studies with a 6‐ to 12‐month follow‐up, the application of a FGG over a partial‐thickness vascular bed represents the gold standard for obtaining KMW gains. The use of a substitute normally renders inferior outcomes, and it should only be used in the presence of a surrounding band of keratinized mucosa.
3. What is the clinical performance of autogenous soft tissue grafts compared with substitute materials in terms of peri‐implant STH augmentation?
  In sites presenting insufficient STH, STA may be considered provided the implant apico‐coronal position is adequate. Although the scientific evidence from RCTs identified in the systematic review is very limited,^5^ the experts’ opinions favor using a subepithelial CTG, although substitutes may also be used for STH augmentation in some specific circumstances.
4. What is the clinical performance of autogenous soft tissue grafts compared with substitute materials for the purpose of correcting PSTD?
  In sites presenting PSTDs and in the absence of peri‐implantitis, STA may be considered provided the implant position and the integrity of the restorative interface are favorable. Although the scientific evidence from RCTs identified in the systematic review is very limited,^5^ the experts’ opinion is in favor of recommending surgical interventions consisting of a bilaminar approach and an autogenous CTG. These interventions are often performed in the context of interdisciplinary therapy (e.g., restorative and/or orthodontic). No RCTs on the use of soft tissue graft substitutes for the treatment of PSTDs were identified.
5. Does the timing of STA relative to implant placement (simultaneous with or after implant placement) and implant placement protocol (immediate vs. delayed) influence the outcomes of therapy?
  The timing of STA and implant placement protocol have not been shown to influence the short‐term outcomes of therapy.
6. Does the source of autogenous CTGs influence the outcomes?
  Intraoral sources of CTGs include the palatal mucosa, tuberosity, and the retromolar pad. Esthetic complications from suboptimal tissue integration (e.g., texture and color) and excessive tissue enlargement over time can result from the use of grafts obtained from any source. However, grafts from the palatal mucosa are typically associated with lower risk.
7. Does material (autogenous grafts vs. substitute) and selection of the surgical approach influence clinician‐reported esthetic outcomes (e.g., tissue color and contour) following peri‐implant STA?
  Based on the qualitative assessment of 20 studies, which were identified in the systematic review,^5^ the use of either autogenous grafts or acellular dermal matrices results in similar short‐term esthetic outcomes when used for MT or STH augmentation. However, for KMW augmentation purposes, the application of a bilayered collagen matrix surrounded by keratinized mucosa (i.e., strip technique) results in superior esthetic outcomes compared to the use of an autogenous FGG.^38^
8. Does material selection (autogenous grafts vs. substitute) influence PROs following peri‐implant STA?
  The use of soft‐tissue graft substitutes eliminates the need for a donor site, reducing surgical time and postoperative morbidity and thereby improving the overall patient experience. The esthetic perception of patients who underwent STA did not significantly differ between those who received autogenous CTG or a substitute scaffold. However, compared with autogenous soft tissue grafts, the use of substitute scaffolds is consistently associated with lower postoperative discomfort. Based on the information reported in the selected literature, postoperative complications (transient donor site bleeding, excessive postoperative edema, premature wound dehiscence, flap or graft necrosis, early implant failure) following peri‐implant STA were rare, as documented in only 10 articles (25%).^39^, ^40^, ^41^, ^42^, ^43^, ^44^, ^45^, ^46^, ^47^, ^48^
9. What other key aspects should be taken into consideration in clinical decision making?
  The characteristics of the recipient site (e.g., existing MT and KMW, implant position, extent of the deformity) and the therapeutic goal(s) should be accounted for in terms of material and surgical approach selection.
  Obtaining an autogenous soft tissue graft from intraoral donor sites may not be feasible or limited due to a variety of reasons (e.g., anatomical, medical, behavioral, and/or patient preferences), which may justify the use of alternatives.
  From a clinical cost perspective, the use of soft tissue graft substitutes involves additional expenses while less chairside time is needed.
  Surgical team/operator experience and level of training also play a key role on case selection and the outcomes of therapy.

### 3.2 Efficacy of cells and/or biologics combined with scaffolds for hard and soft tissue augmentation in implant therapy

3.2.1

As noted in the general introduction, current strategies for bone and soft‐tissue regeneration are invasive, unpredictable, and often fail to reliably restore function and architecture in large defects, particularly in elderly or medically compromised patients, leaving clinicians with limited options and patients without effective, patient‐friendly solutions. These limitations have increased interest in tissue engineering strategies, primarily involving biologics and cell therapies that confer inductive properties to support immune modulation, stimulate proliferation, recruit and differentiate progenitor cells, and promote neoangiogenesis. This WG's consensus report, based on two systematic reviews, aims to evaluate the scientific literature on the use of biologics and cell therapies in combination with other bioengineered approaches for effective intraoral bone and soft tissue regeneration.

These two systematic reviews, prepared specifically for this consensus, thoroughly examined these approaches. Monje et al. evaluated the use of cells and/or biologics combined with scaffolds for alveolar bone regeneration, reporting that biologics and cell therapy combined with scaffolds for ridge augmentation and preservation procedures may yield better outcomes in terms of dimensional gain, as well as histological new bone formation and mineralization, with both techniques being safe and effective in alveolar bone regeneration.^49^ In another systematic review, Montero et al. assessed the use of cells and/or biologics combined with scaffolds for soft‐tissue regeneration at implant sites and reported a lack of evidence for cell‐based therapies or other biologics in this context.^50^

##### Definitions

*Biologics* are naturally derived or recombinant biological products used to promote tissue healing and regeneration. They primarily act as signaling molecules, such as growth factors, cytokines, and peptides, which can be delivered alone or in combination with scaffolds. Biologics can generally stimulate cell proliferation, cell differentiation, angiogenesis, and matrix deposition.

The following biologics have been utilized in hard and soft tissue augmentation in the context of implant therapy:

- *Signaling Molecules* are biologically active compounds, usually growth factors, cytokines, or chemokines, that control cellular behavior by binding to specific receptors and activating downstream signaling pathways. Signaling molecules function as individual agents with targeted biological effects.
- *Morphogens* are a subgroup of signaling molecules that control cell fate and differentiation, guiding the development of specific tissues.
- *Mitogens* are a class of signaling molecules that primarily stimulate cell recruitment, chemotaxis, and proliferation. In periodontal and peri‐implant regeneration, mitogens stimulate fibroblast, osteoprogenitor, and endothelial cell replication, thereby increasing the number of cells available for tissue repair.
- *Recombinant Platelet‐Derived Growth Factor (rhPDGF‐BB)* promotes soft‐ and hard‐tissue regeneration by significantly influencing cell migration, proliferation, differentiation, and angiogenesis. It binds to specific cell‐surface receptors to initiate the wound‐healing process. It has been used with scaffolds for ridge augmentation and preservation, demonstrating enhanced clinical and dimensional outcomes.
- *Recombinant Bone Morphogenetic Proteins (rhBMPs)* stimulate mesenchymal stem cells (MSCs) to differentiate into bone‐forming osteoblasts and cartilage‐forming chondrocytes. BMPs activate gene transcription through both the canonical (Smad‐dependent) and non‐canonical (Smad‐independent) pathways. They have been used with scaffolds for ridge augmentation and ridge preservation, demonstrating enhanced clinical and dimensional outcomes.
- *Enamel Matrix Derivatives (EMDs)* support hard tissue regeneration by enhancing the migration and proliferation of mesenchymal stem cells and preventing epithelial cell ingrowth. EMD also promotes neovascularization by inducing VEGF and helps modulate inflammation.
- *Autologous Blood Products (ABPs)*. ABPs contain a “cocktail” of growth factors, which may or may not be embedded in a fibrin network (depending on preparation). Each can improve hard‐tissue regeneration. Briefly, the main active growth factors are listed below:
  - *Platelet‐Derived Growth Factor (PDGF)*: A powerful chemoattractant that draws in mesenchymal stem cells, fibroblasts, and osteoblasts to the surgical area. It promotes cell growth, collagen production, and the development of new bone matrix.
  - *Transforming Growth Factor‐Beta (TGF‐β)*: Regulates the proliferation and differentiation of progenitor cells and can induce MSCs to become osteoblasts. It also inhibits osteoclast differentiation.
  - *Vascular Endothelial Growth Factor (VEGF)*: A crucial mediator of angiogenesis and neovascularization.
  - *Insulin‐Like Growth Factor‐1 (IGF‐1)*: Stimulates osteoblasts and osteoblast progenitor cells, promotes matrix synthesis, and inhibits osteoblast apoptosis.
  - *Bone Morphogenetic Proteins (BMPs): Same functions as recombinant BMPs above*.
- *Cell Therapy* is the therapeutic use of living cells in the patient's body to treat or prevent disease by promoting tissue regeneration. These include progenitor and stem cells derived from the patient themselves (autologous) or from a donor (allogenic), applied either as expanded cell populations or minimally manipulated tissue fractions. Depending on the source, MSCs are categorized as:
  - *Bone Marrow‐Derived Mesenchymal Stem Cells (BMMSCs)*. These MSCs are extracted explicitly from bone marrow, typically from the iliac crest (hip bone), although they can also be aspirated from other sites, such as the sternum or femur. They are multipotent, meaning they can differentiate into various cell lineages, including osteoblasts (bone cells), chondrocytes (cartilage cells), adipocytes (fat cells), and potentially myocytes and neurons. Beyond their differentiation capabilities, BMMSCs exhibit paracrine functions, including the secretion of growth factors, cytokines, and extracellular vesicles (EVs), thereby promoting tissue repair, modulating immune responses, and reducing inflammation.
  - *Autologous Adipose‐Derived Mesenchymal Stem Cells (ADSCs)* enhance oral hard tissue regeneration through paracrine signaling, secreting growth factors that stimulate osteogenic differentiation and angiogenesis, and immunomodulating the inflammatory response to create a more regenerative environment. Although direct differentiation into bone‐forming cells can occur, the main mechanism is ADSCs' ability to modulate the microenvironment by releasing soluble factors that support the activity of endogenous repair cells and promote tissue‐specific regeneration.
  - *Periosteal‐Derived Stem Cells (P‐SSCs)* can differentiate directly into osteoblasts and chondrocytes and exert paracrine effects by secreting growth factors and other bioactive molecules.
  - *Periodontal Ligament‐Derived Mesenchymal Stem Cells (PDL‐MSCs)* promote tissue regeneration through paracrine effects, releasing EVs and growth factors that regulate inflammation, stimulate cell growth and differentiation (e.g., osteogenesis), and promote angiogenesis. Key secreted factors include microRNAs (miRNAs), pro‐ and anti‐inflammatory cytokines, and growth factors, which help create a favorable local environment for regeneration.
  - *Stem Cells from Human Exfoliated Deciduous Teeth (SHEDs)* demonstrate multiple mechanisms of action, including paracrine effects to promote tissue repair, differentiation into various cell types such as neural cells, odontoblasts, and adipocytes, and immunomodulatory actions to reduce inflammation and activate endogenous healing processes.
  - *Dental Follicle Stem Cells (DFSCs)*, originating from the neural crest, harbor a multipotential differentiation capacity, including the ability to differentiate into osteoblasts, adipocytes, chondrocytes, cementoblasts, periodontal ligament cells, as well as neuronal cells.
  - *Dental Pulp‐Derived Stem Cells (DPSCs)* exert immunomodulatory, regenerative, and neuroprotective effects by differentiating into various cell types and secreting growth factors, cytokines, and EVs. DPSCs regulate the immune system by shifting macrophages from a pro‐inflammatory M1 state to an anti‐inflammatory M2 state and modulating T and B cells, thereby reducing inflammation. For regeneration, they promote neovascularization and angiogenesis, enhance extracellular matrix formation and organization, and secrete factors to stimulate tissue repair. They also release neurotrophic factors and migrate to damaged areas to form new neural tissue.
  - *Tooth Germ Progenitor Stem Cells (TGPSCs)* exhibit multipotency, differentiating into cementoblasts, PDL cells, and osteoblasts, thereby contributing to periodontium development and tooth eruption. TGPSCs also exhibit paracrine effects, secreting factors that modulate the microenvironment and facilitate immunomodulation and tissue repair.
- *Whole Tissue Fractions* offer cell‐based regenerative therapies for oral tissues by delivering a combination of stem cells and other reparative cells, along with their secreted factors. Such therapies work through paracrine signaling, releasing growth factors and cytokines that promote neovascularization and angiogenesis, as well as immunomodulation and the recruitment and chemotaxis of endogenous cells to repair and regenerate oral tissues.
- *Exosome/Secretome Therapy (Cell‐Free)* uses EVs (exosomes, microvesicles) and soluble factors secreted by living cells—most commonly mesenchymal stem/stromal cells (MSCs)—to enhance tissue regeneration. These cell‐free biological products contain proteins, cytokines, growth factors, and regulatory RNAs that mediate many of the paracrine effects of MSCs. They can be derived from either autologous or allogenic MSCs, stored and supplied as ‘off‐the‐shelf’ products, and are considered to have a lower risk of immune rejection or tumor formation than live cell transplantation.

#### 3.2.2 Efficacy of cells and/or biologics combined with scaffolds for hard tissue augmentation in implant therapy

This consensus report was based on the answer to the following questions:

1. What is the effectiveness of biologics combined with scaffolds in terms of dimensional changes for ridge augmentation procedures when compared to control groups?
  The scientific evidence on their efficacy, as reported in the systematic review,^49^ did not allow for a subset analysis of the individual biologics (rhBMP‐2, ABP, rhPDGF‐BB) due to the high heterogeneity of the included studies. When considering the combined effect of the biologics used with scaffolds, it showed a statistically significant but limited effect (*n* = 5; weighted mean difference [WMD] = 0.30 mm) in increasing ridge width for ridge augmentation procedures.^51^, ^52^, ^53^, ^54^, ^55^ The corresponding effect for gaining ridge height was also limited (*n* = 2; WMD = 0.39 mm) and not statistically significant when compared with the control group.^51^, ^56^ Given the small dimensional changes in bone gain attained, the clinical added value of using biologics combined with scaffolds is questionable.
  This effect was assessed before implant placement. Therefore, the long‐term effectiveness of biologics combined with scaffolds still needs to be investigated.
  Similarly, the impact of different scaffolding materials on the effect of added biologics could not be explored due to the high heterogeneity of the included studies. Still, their demonstrated effect is only applicable when using the principle of GBR, since all the included studies used resorbable or non‐resorbable barrier membranes and particulate bone graft materials. None of the included studies evaluated the use of biologics combined with volume‐stable (blocks) devices.
  First‐generation autogenous blood‐derived products (platelet‐rich plasma) showed superiority (*n* = 2; WMD = 0.52 mm) in enhancing ridge width.^51^, ^54^
2. What is the effectiveness of biologics combined with scaffolds in terms of histological outcomes for ridge augmentation procedures?
  Due to the limited human histological evidence, the effectiveness of added biologics based on histological outcomes could not be assessed.
3. What are the reported adverse events and complications when using biologics combined with scaffolds for ridge augmentation procedures?
  There were no major adverse events or complications (*n* = 4) when using biologics combined with scaffolds.^51^, ^55^, ^57^, ^58^ Only minor complications have been reported, particularly with the use of rhBMP‐2, which was associated with facial swelling (*n* = 1).^55^
4. What are the reported PROs and ClinROs when using biologics combined with scaffolds for ridge augmentation?
  There were no severe adverse PROs reported (*n* = 2) when using biologics combined with scaffolds.^58^, ^59^ Patients reported an increased willingness to repeat surgery and reduced postoperative discomfort when platelet‐rich fibrin (PRF) was combined with scaffolds (*n* = 1).^59^ Conversely, intra‐ and extra‐oral sensitivity at suture removal was associated with the use of rhBMP‐2 (*n* = 1).^59^
5. What is the effectiveness of biologics combined with scaffolds in terms of dimensional changes for ridge preservation procedures?
  When considering the combined effect of biologics (EMD, rhBMP‐2, ABP, rhPDGF) used with scaffolds, a statistically significant but limited effect was observed (*n* = 7; WMD = 0.48 mm) in attenuating ridge width reduction.^60^, ^61^, ^62^, ^63^, ^64^, ^65^, ^66^ The corresponding effect in reducing ridge height change was also limited (*n* = 6; WMD = 0.40 mm).^60^, ^61^, ^62^, ^63^, ^64^, ^65^ Given the small dimensional changes in bone gain achieved when comparing test and control groups, the clinical added value of using biologics with scaffolds is questionable in ridge preservation interventions. Furthermore, this effect was always assessed before implant placement. Therefore, the long‐term effectiveness of biologics in combination with scaffolds still requires further investigation.
  rhBMP‐2 (1.50 mg/mL) showed superiority (*n* = 3; WMD = 1.08 mm) in preventing ridge width resorption.^62^, ^63^, ^64^ Similarly, the impact of different scaffolding materials on the effect of added biologics could not be explored due to the high heterogeneity of the included studies. When used without scaffolds, ABPs demonstrated effectiveness for ridge preservation compared to spontaneous socket healing in one RCT.^67^ However, these findings were not confirmed in another RCT that showed similar results when comparing PRF with unassisted socket healing.^67^ These studies were excluded from the systematic review because the biologics tested were not combined with scaffolds.
6. What is the effectiveness of biologics combined with scaffolds in terms of histological outcomes for ridge preservation procedures?
  There is limited human histological evidence. In two studies, the use of biologics resulted in less non‐mineralized tissue at 4 months (*n* = 2; WMD = −14.9%).^61^, ^68^
7. What are the reported adverse events and complications when using biologics combined with scaffolds for ridge preservation procedures?
  There were no major or minor adverse events or complications (*n* = 3) when using biologics combined with scaffolds in ridge preservation interventions.^64^, ^69^, ^70^
8. What are the reported PROs and ClinROs when using biologics combined with scaffolds for ridge preservation procedures?
  There were no adverse PROs reported (*n* = 2) when using biologics combined with scaffolds.^65^, ^70^ Patients reported a willingness to undergo repeat surgery and reduced postoperative discomfort when PRF was combined with scaffolds (*n* = 1).^70^
9. What is the effectiveness of biologics combined with scaffolds in terms of dimensional changes for maxillary sinus floor elevation procedures?
  The scientific evidence on their efficacy, as reported in the systematic review,^49^ did not allow for a subset analysis of the individual biologics (EMD, rhBMP‐2, ABP) due to the high heterogeneity of the included studies. When considering the combined effect of the biologics used with scaffolds, a statistically significant, but limited effect (*n* = 2; WMD = 0.32 mm) was observed in increasing vertical bone height.^71^, ^72^ Given the small dimensional changes in bone gain observed when comparing test and control groups, the clinical added value of using biologics with scaffolds in sinus floor elevation procedures is questionable, and this effect was always assessed before implant placement. Therefore, the long‐term effectiveness of biologics in combination with scaffolds still requires further investigation.
  Similarly, the impact of different scaffolding materials on the effect of added biologics could not be explored due to the high heterogeneity of the included studies.
10. What is the effectiveness of biologics combined with scaffolds in terms of histological outcomes for maxillary sinus floor elevation procedures?
  There is limited human histological evidence. In five studies, the use of biologics resulted in less residual graft particles at (≤6 months) (*n* = 5; WMD = −5.54%).^73^, ^74^, ^75^, ^76^, ^77^
11. What are the reported adverse events and complications when used biologics combined with scaffolds for maxillary sinus floor elevation procedures?
  There were no major or minor adverse events or complications (*n* = 1) when using biologics combined with scaffolds in sinus floor elevation procedures. Use of higher dosage (1.50 mg/mL) versus (0.75 mg/mL) rhBMP‐2 (*n* = 1) was associated with higher edema but lesser pain.^72^
12. What are the reported PROs and ClinROs when using biologics combined with scaffolds for maxillary sinus floor elevation procedures?
  No adverse PROs were reported (*n* = 1) when using biologics combined with scaffolds. Patients reported an increased willingness to repeat surgery when PRF was combined with scaffolds (*n* = 1).^78^
13. What is the impact of the use of biologics combined with scaffolds to implant‐related outcomes?
  Evidence from clinical studies suggested no differences in implant stability quotient during implant placement (*n* = 1),^79^ marginal bone levels (*n* = 2),^79^, ^80^ or clinical peri‐implant parameters (*n* = 1).^80^
14. What is the effectiveness of cell therapies combined with scaffolds in terms of dimensional changes for ridge augmentation procedures when compared to control groups?
  One recent multicenter study using BMMSCs demonstrated statistically significant volumetric changes with scaffold‐based cell therapy compared with standardized therapy (autogenous block graft) at 5 months postoperatively (mean difference of 480.01 mm^3^).^81^ Other studies included in this systematic review do not show statistically significant differences in dimensional changes with the use of cell therapies and scaffolds^82^, ^83^ compared to scaffolds alone.
15. What is the effectiveness of cell therapies combined with scaffolds in terms of histological changes for ridge augmentation procedures when compared to control groups?
  No data were available from the included studies in this systematic review to demonstrate differences in histologic findings with the inclusion of cell therapies and scaffolds compared to scaffolds alone for ridge augmentation procedures.
16. What is the effectiveness of cell therapies combined with scaffolds in terms of PROs for ridge augmentation procedures when compared to control groups?
  Only minor or no adverse events were reported with the use of cell therapies, and therefore, these treatments were regarded as safe for ridge augmentation.^84^ No data from the included studies in this systematic review demonstrated differences in PROs between cell therapies combined with scaffolds and scaffolds alone for ridge augmentation procedures.
17. What is the effectiveness of cell therapies combined with scaffolds in terms of dimensional changes for ridge preservation procedures when compared to control groups?
  One study using BMMSCs included in the systematic review demonstrated that the addition of cell therapies with scaffolds resulted in statistically superior acceleration of radiographic linear bone height within the extraction defect (mean difference 23.6%) compared to scaffold alone at the 6‐week time point, but not at 12 weeks postoperatively (mean difference 5.4%).^85^
18. What is the effectiveness of cell therapies combined with scaffolds in terms of histological changes for ridge preservation procedures when compared to control groups?
  One study using BMMSCs included in the systematic review showed that the use of cell therapies with scaffolds increased bone volume fraction and bone mineral density in microCT analysis compared to scaffold use alone (mean bone volume fraction difference 0.15; mean bone mineral density difference 109.5 mg/cc). However, these changes in microCT outcomes were not significant at 12 weeks postoperatively.^85^
19. What is the effectiveness of cell therapies combined with scaffolds in terms of PROs for ridge preservation procedures when compared to control groups?
  No adverse events were reported with the use of cell therapies, and the treatments were considered safe for use in humans for ridge preservation.^64^, ^69^, ^70^ No data were available from the included studies in this systematic review to show differences in PROs with the inclusion of cell therapies and scaffolds compared to scaffolds alone for ridge preservation procedures.
20. What is the effectiveness of cell therapies combined with scaffolds in terms of dimensional changes for sinus floor elevation procedures when compared to control groups?
  In the studies included in this systematic review, no differences in radiographic bone height or dimensional changes were observed between cell therapies with scaffolds and scaffolds alone.^86^, ^87^, ^88^, ^89^
21. What is the effectiveness of cell therapies combined with scaffolds in terms of histological changes for sinus floor elevation procedures when compared to control groups?
  One study using whole tissue fraction cells demonstrated a higher percentage of newly formed bone in histologic analysis during sinus floor elevation procedures when cell therapies with scaffolds were used compared to scaffolds alone (63.18% more bone formation in the cell therapy group)^90^ Other studies showed no significant differences in histologic findings between sinus floor elevation procedures using cell therapies with scaffolds and those using scaffolds alone.^88^, ^91^
22. What is the effectiveness of cell therapies combined with scaffolds in terms of PROs for sinus floor elevation procedures when compared to control groups?
  One study using whole tissue fraction cells reported that there were no adverse events linked to cell therapies, and the treatments were generally considered safe for human use during sinus floor elevation procedures.^88^ In one study, patients reported a willingness to undergo the procedure again, along with reported PROs.^88^ while in another study, patients reported high overall satisfaction.^91^ No data were available from the studies included in this systematic review to demonstrate differences in PROs when comparing cell therapies and scaffolds to scaffolds alone for sinus floor elevation procedures.
23. What other application of cell therapies combined with scaffolds have been reported in the literature, not covered in the systematic review.
  MSCs therapies have also shown usefulness for conditions beyond those covered in the systematic review, such as cyst‐associated defects,^92^ cleft palate,^93^ and non‐union fractures.^94^ They demonstrate effectiveness in promoting hard tissue regeneration, improving perioperative safety, and have no significant adverse events.
  Regarding the effectiveness of cell therapies for these indications, results are variable across different clinical scenarios. Alveolar bone‐derived MSCs transplanted into bone defects after removing cystic lesions, combined with autologous serum cross‐linked matrices, showed increased CT density compared to the untreated control group at 7 months post‐treatment.^92^ Similarly, cell therapies may benefit individuals with non‐union fractures, with bone healing rates reaching 91% at 12 months postoperatively and significantly shorter bone union times compared to bone grafts alone (SMD: −0.54 months) according to a recent systematic review.^95^ However, treating large cleft palate defects with ADSCs and BMMSCs did not show superiority to autologous bone block grafts.^93^, ^94^
24. Which emerging cell therapy technologies have the highest potential for hard tissue regeneration interventions?
  Both expanded MSCs and whole tissue fractions appear to be safe and may offer additional benefits for increased volumetric bone gain, early bone turnover, and new bone formation. These therapies could be particularly advantageous in non‐contained bony defects and for individuals who may benefit from enhanced healing, such as those with impaired wound healing or cell senescence. It is also important to note that a better understanding of the utility of cell therapies could be improved by using consistent scaffolds throughout treatments, and evaluating cell therapies without scaffolds was outside the scope of this systematic review. When considering the clinical application of cell therapies, cost‐utility analyses could help clinicians make more informed decisions about incorporating these treatments into their practice. Additionally, cell therapies derived from other sources—such as PDL‐MSCs, SHEDs, DFSCs, DPSCs, TGPSCs, and whole tissue fractions—may offer further benefits by enhancing cell recruitment, differentiation, and tissue regeneration. Emerging therapies also include autologous and allogenic stem cell treatments combined with customized scaffolds, such as hydrogels, printed frameworks, and biomimetic structures.

#### 3.2.3 Efficacy of cells and/or biologics combined with scaffolds for soft tissue augmentation in implant therapy

Soft tissue augmentation is recommended for soft tissue deficiencies at dental implant sites that may affect long‐term peri‐implant tissue health or aesthetics. The main objectives are to increase (i) peri‐implant soft tissue thickness (STT), (ii) the width of peri‐implant keratinized mucosa (PIKM), or (iii) to address mucosal dehiscence at dental implant sites.

The standard of care treatments typically rely on autogenous soft tissue grafts, such as free gingival grafts (FGGs) and CTGs. The FGG is considered the gold standard for increasing PIKM width,^38^ while bilaminar techniques using CTGs are regarded as the most predictable methods to augment peri‐implant MT and to correct mucosal dehiscences.^8^, ^96^ Despite their predictability, autogenous grafts are linked to donor site morbidity, limited availability, and longer surgical times. To address these issues, soft tissue substitutes (e.g., xenogeneic collagen matrices, acellular dermal matrices of xenogeneic or allogenic origin (ADM) have been introduced as alternatives or adjuncts. They decrease morbidity and surgical time, and systematic reviews verify their effectiveness, although autogenous grafts still offer superior results in terms of STT, PIKM gain, and long‐term stability.^8^, ^38^, ^96^ Therefore, the current standard of care remains autogenous grafting, while substitutes are increasingly used in cases where patient preference, morbidity reduction, or anatomical/medical conditions limit harvesting.

The biologics studied include autologous blood‐derived products, especially various forms of platelet concentrates, mainly PRF, including leukocyte PRF (L‐PRF) and titanium‐prepared PRF (T‐PRF). Other biologics include rhPDGF‐BB, amnion/chorion allograft membranes, and hyaluronic acid. EMDs have been evaluated at dental implant sites in preclinical in vivo studies.^97^

Investigated cell therapies include the use of micronized‐gingival connective tissue (MGCT), which provides gingival mucosa‐derived mesenchymal stem cells.^98^ Living cellular constructs (such as fibroblasts and keratinocytes on different scaffolds) and fibroblast‐seeded soft‐tissue substitutes have been studied around teeth.^99^, ^100^

The consensus report was based on the answer to the following questions:

1. Which biologics have demonstrated efficacy in improving outcomes when used alone or with a scaffold in: (a) increasing soft tissue thickness, (b) augmenting keratinized mucosa, and (c) covering PSTD defects?
  - *Increasing STT*:
    Up to 6 months, meta‐analyses showed a weighted mean effect (WME) of a 1.5 mm increase in STT at the midfacial mucosal level (*n* = 4)^41^, ^101^, ^102^, ^103^ a 1.3 mm increase at the occlusal level (*n* = 4),^101^, ^102^, ^103^, ^104^ and a 0.5 mm increase beyond the mucogingival line (*n* = 2) with the use of either PRF membranes or rhPDGF‐BB‐soaked collagen matrices.^102^, ^103^
    PRF membranes, when placed around healing abutments, have been shown to significantly increase STT (ranging from 0.9 to 2.8 mm at the midfacial mucosal level) compared to no intervention.^41^, ^101^ A prospective case series evaluating a collagen matrix infused with rhPDGF‐BB also demonstrated a moderate increase (2.14 ± 3.27 mm) in STT at 4 months.^102^
  - *Augmenting PIKM*:
    Compared to FGGs, the use of PRF membranes resulted in inferior outcomes up to 3 months (*n* = 2; WMD = 2.0 mm in favor of FGG).^105^, ^106^ However, when compared to no attempt to increase STT, PRF membranes significantly increase PIKM width (*n* = 2; WMD = 1.1 mm).^41^, ^101^ Amnion/chorion allograft membranes showed a significant increase in PIKM in one prospective case series (2 mm [95% confidence interval: 1.4–2.6]) at 60 days.^107^
  - *Covering PSTD defects*:
    There is no scientific evidence regarding the use of biologics in combination with scaffolds for treating PSTD defects.
2. Which cell therapies have demonstrated efficacy in improving outcomes when used with a scaffold in: (a) increasing soft tissue thickness or augmenting keratinized mucosa, and (b) covering PSTD defects?
  - *Increasing STT and augmenting PIKM*:
    There is no scientific evidence supporting the use of cell therapies combined with scaffolds to increase STT or PIKM. However, in one pilot case series, the use of MGCTs with an atelocollagen matrix showed preliminary effectiveness in regenerating peri‐implant mucosa, but specific gains in STT or PIKM were not quantified.^98^
  - *Covering PSTD Defects*:
    There is no scientific evidence on the use of cell therapies together with scaffolds for the treatment of PSTD defects.
3. Which are the biologics with demonstrated histological efficacy in soft tissue regeneration at implant sites?
  None of the included studies provided human histological data. Preclinical in vivo experimental investigations suggest that EMD can enhance angiogenesis, fibroblast proliferation, and collagen fiber orientation around implants, supporting soft tissue maturation.^97^ Similarly, in vivo experimental studies show increased vascular density, fibroblast proliferation, and faster re‐epithelialization when using PRF on teeth.^108^ Furthermore, there is promising data on the use of polydeoxyribonucleotide [PDRN]‐loaded collagen matrices, demonstrating PIKM formation comparable to FGG both clinically and histologically in in vivo experimental studies.^109^
4. Which are the cell therapies with demonstrated histological efficacy in soft tissue regeneration at implant sites?
  None of the included studies provided human histological data. Preclinical in vivo experimental studies have shown that MSCs can home to peri‐implant mucosal sites and enhance soft tissue healing, such as reinforcement of epithelial sealing around implants.^110^ Additionally, histological evaluation in in vivo experimental peri‐implant models indicate that fibroblast‐seeded matrices integrate with host tissue, showing keratinized epithelium and connective tissue organization similar to FGG.^111^
5. Is the use of biologics safe as an adjunct to other soft tissue regenerative interventions, and what is the impact of their use on PROs?
  The use of the evaluated biologics (i.e., L‐PRF, rhPDGF‐BB) is considered safe. Regarding PROs, the use of L‐PRF in combination with an apically positioned flap, when compared to a FGG, resulted in significantly less postoperative pain and discomfort during the first 6 days after surgery.^105^
6. Is the use of cell therapies safe as an adjunct to other soft tissue regenerative treatments, and what is their effect on PROs?
  There is no scientific evidence of cell therapy on PROs. However, a pilot case series using MGCTs found the technique to be safe.^98^
7. Which emerging technologies in biologics have the highest potential in peri‐implant soft tissue regeneration interventions?
  Evidence from the systematic review indicates a need for further research into biologics beyond PRF, used with or without soft tissue substitutes (e.g., growth‐factor‐loaded matrices) and/or hyaluronic acid (HA) ‐based injectables or other bioactive hydrogels as delivery platforms. Among emerging biologics, polynucleotides, miRNAs, and EVs may influence inflammation, angiogenesis, and fibroblast activity without the regulatory and viability challenges associated with living cells.
8. Which emerging cell therapy technologies have the highest potential in peri‐implant soft tissue regeneration interventions?
  Preclinical studies on 3D‐bioprinted scaffolds seeded with gingival fibroblasts have shown promising results for improving keratinized tissue augmentation,^112^ but there is a need for human studies specifically focused on their role at dental implant sites. Emerging cell therapies for peri‐implant soft tissue regeneration include oral mucosa/gingival cell‐sheets,^113^ gingival mesenchymal stem cell‐based therapies,^114^ and tissue‐engineered oral mucosa equivalents.^115^

### 3.3 Current, emerging, and personalized approaches for periodontal and peri‐implant tissue regeneration

#### Definitions

- *Site‐Specific Approach*: Treatment modalities involving customized defect‐specific fabrication utilizing digital image‐based planning data and computer‐aided design and manufacturing technologies.
- *Precision Periodontal Medicine*: Oral health care designed to optimize efficiency or therapeutic benefit for individual groups of periodontal patients. In addition, profiling by genetic, molecular, microbiome is used in combination with clinical information and patient demographics.
- *Minimally Invasive*: Interventions designed to enhance tissue quantity or quality by avoiding or minimizing incisions, flap elevation, and/or use of autogenous tissue. This aims to minimize surgical trauma, preserve vascular integrity, and promote rapid, esthetically favorable healing, pursuing less patient discomfort and morbidity.
- *Emerging Technologies*: Novel approaches, for which preliminary evidence is available suggesting their promise in oral tissue regeneration.

The consensus report was based on the answer to the following questions:

1. What is the relative tissue thickness that can be achieved at tooth and implant sites?
  The increase in soft tissue thickness is higher around implants (0.68–1.12 mm) than teeth (0.33–0.62 mm). It is acknowledged soft tissue thickening around teeth was undertaken in conjunction with root coverage, which was not the case around implants.
2. What are the most effective materials for increasing soft tissue thickness at tooth and implant sites?
  At natural teeth, all the explored treatment modalities led to a significant increase in gingival thickness compared to no augmentation (flap treatments alone), with CTG showing the highest gain (0.62 mm), followed by volume‐stable collagen matrices (VCMX) (0.57 mm), hADM (0.48 mm), pADM (0.47 mm), and collagen matrices (CMX) (0.33 mm). At implant sites, the most effective treatment was the CTG (1.12 mm), followed by hADM (0.98 mm), VCMX (0.88 mm), pADM (0.71 mm), and CMX (0.68 mm). Different surgical techniques were used in these studies, but no comparative data is available. The reported results are based on type of material, not accounting for different surgical techniques.
3. Is there a difference in PROs using different approaches to soft tissue thickness augmentation?
  There are measurable differences in PROs between autogenous CTG and soft‐tissue substitutes (e.g., collagen matrices, ADM, VCMX). Soft‐tissue substitutes reduce donor‐site morbidity, operative time, and postoperative pain, and often give similar patient satisfaction and esthetic PROs, despite slightly smaller tissue gains.^116^, ^117^, ^118^
4. What are the long‐term outcomes of augmentation procedures for soft tissue thickening at tooth and implant sites?
  On natural teeth, long‐term stability has been documented with a 9‐ and 12‐year follow‐up for hADM and autogenous CTGs, respectively.^119^, ^120^, ^121^, ^122^, ^123^ At dental implant sites, stable outcomes have been reported with a follow‐up of 3–12 years.^8^, ^124^, ^125^, ^126^, ^127^
5. What are the fabrication techniques for personalized site‐specific scaffolds?
  Subtractive techniques (e.g., milling) are used for the manufacturing of titanium meshes or plates, custom allogenic or ceramic blocks.^83^ Their advantages include dimensional accuracy, compatibility with existing biomaterials.^83^, ^94^, ^128^ Limitations include higher material waste, and challenges for complex internal geometries and imparting porosity. Additive techniques (e.g., 3D printing) include selective laser melting and electron beam melting for metals; and extrusion, fused deposition modeling, stereolithography, and digital light processing for polymers and ceramics.^86^, ^129^, ^130^, ^131^ Among these techniques, the following applications have been tested: custom titanium meshes, printed ceramic scaffolds, thermoplastic polymers, and guides for positioning or fixation. Their main advantage is the ability to achieve controllable porosity and geometric freedom for complex architectures. Among the limitations: variable clinical validation and quality control dependent on the printer and process.
6. What are the biomaterials used in personalized site‐specific scaffolds for oral tissue regeneration?
  Different biomaterials have been tested:
*Titanium*: Produced as CAD/CAM meshes or plates, these devices have the largest clinical evidence base. Advantages are high rigidity, stability, and ease of fixation. Disadvantages are risk of exposure if soft tissue coverage is compromised, and due to being non‐resorbable, requirement for a second surgery for removal.^94^, ^128^
*Custom allograft blocks*: Advantages are good osteoconduction and precise adaptation. Disadvantages are biological variability, and logistical and regulatory challenges.
*Ceramics* (hydroxyapatite and β‐tricalcium phosphate [β‐TCP]): Produced as 3D‐printed scaffolds with controllable porous architecture, their resorption and integration depend on composition and design. Advantages are good osteoconduction and precise adaptation. Disadvantages are their brittle nature and challenging surgical fixation.^132^, ^133^
*Thermoplastic synthetic polymers (polycaprolactone [PCL]; PEEK)*: Lightweight non‐resorbable (PEEK) or resorbable (PCL) structures used as frames or 3D scaffolds. The main advantage is ready availability, low cost, and ease of manufacturing. Disadvantage is low bioactivity, necessitating their use in combination with bioactive coatings, biomaterials, or biologics.^133^, ^134^
*Composites of ceramics and thermoplastic synthetic polymers (e.g., PCL– β‐TCP)*.^118^ These devices aim to take advantage of potentially synergistic favorable properties of polymers (favorable fabrication and handling) and ceramics (bioactivity), to overcome challenges with pure ceramic (brittleness) and polymer (low bioactivity) devices.
7. What is the efficacy and safety of personalized site‐specific scaffolds for oral tissue regeneration?
  In *soft tissue regeneration*, there are currently no clinically validated, patient‐ or site‐specific soft tissue matrices available.
  In *alveolar bone regeneration*, most clinical evidence pertains to ridge augmentation procedures. Regarding dimensional effectiveness, case series and systematic reviews report mean horizontal gains of approximately 5 mm and vertical gains of 5–6 mm when using personalized image‐based scaffolds, with regenerated volumes ranging from 1.0 to 1.7 cm^3^ at 6–9 months, thereby facilitating implant placement and rehabilitation.^83^, ^86^, ^94^ In terms of safety, complication rates are reported to be approximately 20%–25%, with early complications such as mesh exposure occurring more frequently in the absence of a covering membrane. The outcomes are influenced by factors such as smoking status, operator experience, and defect characteristics.^83^, ^94^ Implant survival rates following successful regeneration are high and appear to depend on effective exposure control and graft stability. Histological analyses demonstrate new bone formation and variable degrees of residual material resorption, depending on the scaffold material and design.^135^, ^136^
  In *periodontal regeneration*, such approaches are emerging, but the available evidence remains limited to clinical case reports.^137^
8. What are the most promising emerging technologies for periodontal and peri‐implant tissue regeneration?
*Composite materials* for enhanced scaffold properties (medical‐grade natural or synthetic polymers combined with ceramics or metal ions)^138^, ^139^
*Resorbable metals* as alternatives to titanium (magnesium, zinc, iron, alloys)^140^
*Stem‐cell based approaches*, including alternative sources (e.g., allogenic)^86^, ^130^, ^131^, ^134^, ^141^
  Bioprinting with cell‐, gene‐, or protein‐laden bioinks^142^, ^143^
*Multiphasic scaffolds* for complex anatomical structures (e.g., periodontium)^144^, ^145^
*Cell‐free extracts* (e.g., secretome, exosomes, EVs).^129^, ^133^ With the potential to help in overcoming regulatory challenges associated with autologous cell therapy. There is promising preclinical data, but clinical data are not available. The main challenges are associated with standardization and manufacturing at scale.
*Immunomodulation* (e.g., resolvins,^146^ bacteriocins^147^).
*Bone anabolic agents* (e.g., abaloparatide, teriparatide, romosozumab)^148^, ^149^
*Total tooth regeneration* (uterine sensitization‐associated gene‐1[USAG‐1] antagonization)^150^
9. What are the existing and emerging roles of artificial intelligence (AI)‐enabled modeling in periodontal and peri‐implant tissue regeneration?
  In *clinical practice*: diagnostic modeling for assisted imaging^151^, ^152^, ^153^, ^154^, ^155^
  In *research*: prediction of peri‐implant regenerative outcomes; prognostic modeling for clinical responses to periodontal treatment^151^, ^156^, ^157^, ^158^
*Emerging*: Deep phenotyping for precision periodontal regenerative medicine; Individual risk assessment based on multimodal data (e.g., multi‐omics, demographics, electronic health records, imaging, social determinants of health)^156^, ^159^

### 3.4 Implications for clinical practice

Patients’ optimal oral health and appropriate medical status should be achieved as a prerequisite for these regenerative procedures. Once planned, these procedures should be discussed with patients, with appropriate emphasis on the indications, risks, benefits, alternatives, and potential complications.

Soft‐ and hard‐tissue regenerative approaches should balance clinical efficacy with patient comfort, esthetic expectations, and anatomical conditions. Particularly in bone regeneration procedures, the incidence of complications may be relevant and impactful; therefore, clinicians should place importance on case selection, surgical training and experience, and proper knowledge and training in managing adverse events.

These procedures should be performed by experienced surgeons using evidence‐based techniques and, when indicated, minimally invasive approaches, since reduced surgical time and minimized trauma are key to success. The digital workflow should be incorporated to align the planned augmentation with implant positioning and prosthetic restoration.

Emerging technologies in bone and soft tissue regeneration are promising for the management of complex defects, and a shift in the focus of AI‐enabled modeling could facilitate patient stratification and the prediction of individual outcomes for precision regenerative treatment.

For soft tissue regeneration, soft tissue substitutes are viable alternatives to autologous grafts, particularly in minimally invasive, patient‐centered care scenarios.

### 3.5 Recommendations for research

The following recommendations address potential areas of research that may enhance the outcomes of new technologies in oral reconstructive medicine:

- Integration of advanced imaging technologies for treatment planning and outcome monitoring, together with the development of AI‐enabled diagnostic and prognostic tools and workflow automation.
- Exploration of robotic surgery approaches in combination with regeneration‐based techniques.
- Generation of validated data by adequately powered RCTs to evaluate meaningful long‐term ClinROs and PROs/PREs, PROs and PREs to capture treatment endpoints. Future studies should also evaluate cost‐effectiveness.
- Incorporate composite outcomes to comprehensively assess the performance of regenerative and reconstructive interventions, including assessment of dimensional changes, in accordance with the Implant Dentistry Core Outcome Set and Measurement (ID‐COSM) recommendations.
- Investigations combining biologics and cell therapies with personalized scaffold constructs may further advance regenerative precision. Additional RCTs are required to validate stem cell‐based and personalized scaffold approaches for bone regeneration. Future research should also integrate AI and digital technologies into clinical workflows for treatment planning and outcome prediction.
- The relevance of personalized bone regeneration approaches should be validated with respect to time, accuracy, stability, histological outcomes, and long‐term results.
- The relevance of soft tissue regenerative approaches should be validated with respect to long‐term stability, histological outcomes, and cost‐effectiveness, while maintaining a careful balance among efficacy, patient morbidity, and satisfaction. Further research should focus on soft‐tissue expanders and papilla‐injection techniques.

### 3.6 Relevance to patients and industry

Patients should be aware of the indications, risks, benefits, alternatives, and complications of the proposed therapies before commencing reconstructive treatment.

The aim of these interventions (bone regeneration and soft tissue grafting) is to regenerate lost or deficient tissues (bone and/or gingivae [gums]) to optimize implant treatment and thereby improve health, function, and esthetics. To achieve these aims, patients should be committed throughout the treatment, placing special emphasis on post‐operative care and long‐term maintenance.

The industry's commitment to future research and development of new technologies, as well as to refining existing ones, is crucial to advancing the field.

## AUTHOR CONTRIBUTIONS

Chairs substantially contributed to the conception and design of the project, to the interpretation of data, and to the drafting and critical review of the manuscript. The Workshop participants made significant contributions by critically reviewing the consensus report and participating in the Workshop discussions. All authors approved the final version of the manuscript.

## CONFLICT OF INTEREST STATEMENT

Participants filed detailed disclosure of potential conflicts of interest relevant to the meeting topic, and these are kept on file. Companies and organizations identified in these disclosures are: American Dental Association, BioHorizons, California Dental Association, CAMLOG, Dentsply Sirona, Geistlich Pharma AG, Mectron S.p.A., Nobel Biocare, OSSTEM Implant Co., Ltd., Osteology Foundation, Purgo Biologics Inc., Straumann Group, Zeiss Medical Technology, and ZimVie Inc.
